# Supplementary material for: Preclinical transmission of prions by blood transfusion is influenced by donor genotype and route of infection
Source: PLoS Pathog. 2021 Feb 18;17(2):e1009276. doi: 10.1371/journal.ppat.1009276 (PMC7891701; doi:10.1371/journal.ppat.1009276)
Supplement: S1 Table — (PDF) [file ppat.1009276.s001.pdf]

S1 Table.

Donor ID	*PRNP* codon 141	Clinical status*	Survival period (dpi) - uninfected donors	Survival period (dpi) - infected donors	Time of donation (dpi)	%SP	IHC - brain	IHC - peripheral tissues**	Western blot - brain	Western blot - peripheral tissues**
**BSE-infected donor; all recipients infected (5 donors)**										
N257	LL	Positive	NA	614	313	51	Positive	Ton + , PP +, Spl +, PLN +, MLN +	Positive	NT
N236	LL	Positive	NA	551	314	57	Positive	Ton + , PP +, Spl +, PLN +, MLN +	Positive	NT
N233	LL	Positive	NA	628	301	48	Positive	Ton + , PP +, Spl +, PLN +, MLN +	Positive	NT
N251	LL	Positive	NA	534	326	61	Positive	Ton + , PP +, Spl +, PLN +, MLN +	Positive	NT
N261	LL	Positive	NA	564	293	52	Positive	Ton + , PP +, Spl +, PLN +, MLN +	Positive	NT
**BSE-infected donor; some recipients infected (10 donors)**										
N178	LF	Positive	NA	1168	315	27	Positive	Ton + , PP -, Spl +, PLN +, MLN +	Positive	Spl +
N245	LF	Positive	NA	959	228	24	Positive	Ton + , PP -, Spl +, PLN +, MLN +	Positive	NT
N189	LL	Positive	NA	586	305	52	Positive	Ton + , PP +, Spl +, PLN +, MLN +	Positive	Spl +, PLN +
N204	LF	Positive	NA	1132	317	28	Positive	Ton + , PP +, Spl +, PLN +, MLN +	Positive	Spl +
N231	LL	Positive	NA	603	314	52	Positive	Ton + , PP +, Spl +, PLN +, MLN +	Positive	NT
N218	FF	Positive	NA	840	378	45	Positive	Ton + , PP +, Spl +, PLN +, MLN +	Positive	NT
N232	LL	Positive	NA	638	332	52	Positive	Ton + , PP +, Spl +, PLN +, MLN +	Positive	NT
N226	FF	Positive	NA	858	326	38	Positive	Ton + , PP +, Spl +, PLN +, MLN -	Positive	Spl +
N157	LL	Positive	NA	602	337	56	Positive	Ton + , PP +, Spl +, PLN +, MLN +	Positive	NT
N180	FF	Positive	NA	817	302	37	Positive	Ton + , PP +, Spl +, PLN +, MLN +	Positive	NT
**BSE-infected donor; no recipients infected (18 donors)**										
N196	LL	Positive	NA	614	289	47	Positive	Ton + , Spl +, PLN -, MLN -	Positive	NT
N206	FF	Intercurrent	NA	835	293	35	Positive	Ton + , PP +, Spl +, PLN +, MLN +	Positive	Spl +, PLN +
N264	LF	Positive	NA	1554	298	19	Positive	Ton + , PP +, Spl +, PLN +, MLN -	Positive	NT
N175	LF	Intercurrent	NA	2002	307	15	Positive	Ton - , PP -, Spl -, PLN -, MLN -	Positive	NT
N246	LF	Positive	NA	1380	293	21	Positive	Ton + , PP -, Spl +, PLN -, MLN -	Positive	NT
N241	LF	Positive	NA	1455	298	20	Positive	Ton - , PP -, Spl +, PLN +, MLN -	Positive	NT
N234	LF	Positive	NA	1092	315	29	Positive	Ton + , PP -, Spl +, PLN -, MLN -	Positive	NT
N223	LF	Positive	NA	1231	291	24	Positive	Ton + , PP +, Spl +, PLN +, MLN -	Positive	NT
N258	LL	Positive	NA	944	293	31	Positive	Ton + , PP +, Spl +, PLN +, MLN +	Positive	NT

N220	FF	Positive	NA	797	298	37	Positive	Ton + , PP +, Spl +, PLN +, MLN +	Positive	Spl +
N188	FF	Positive	NA	664	321	48	Positive	Ton + , PP +, Spl -, PLN +, MLN -	Positive	NT
N161	LF	Positive	NA	1185	294	25	Positive	Ton + , PP +, Spl -, PLN -, MLN -	Positive	NT
N228	FF	Positive	NA	924	299	32	Positive	Ton + , PP +, Spl +, PLN +, MLN -	Positive	Spl +
N164	FF	Positive	NA	1128	315	28	Positive	Ton - , PP +, Spl +, PLN -, MLN -	Positive	NT
N208	LF	Positive	NA	1226	317	26	Positive	Ton + , PP +, Spl +, PLN -, MLN -	Positive	NT
N248	LF	Positive	NA	1242	322	26	Positive	Ton + , PP +, Spl +, PLN +, MLN -	Positive	NT
N259	LF	Positive	NA	1076	333	31	Positive	Ton + , PP +, Spl +, PLN +, MLN +	Positive	Spl +
N169	LF	Positive	NA	1593	300	19	Positive	Ton - , PP +, Spl +, PLN +, MLN -	Positive	NT
**Infection not confirmed in donor; no infected recipients (6 donors)**										
N201	LF	Ataxia	320	NA	291	NA	Negative	Ton - , PP -, Spl -, PLN -, MLN -	Negative	Spl -
N159	LF	Ataxia	683	NA	300	NA	Negative	Ton - , PP -, Spl -, PLN -, MLN -	Negative	NT
N181	FF	Negative	2294	NA	319	NA	Negative	Ton - , PP -, Spl -	Negative	NT
N219	LF	Ataxia	1944	NA	312	NA	Negative	Ton - , PP -, Spl -	Negative	Spl -
N250	LL	Ataxia	258	NA	258	NA	Negative	Ton - , PP -, Spl -, PLN -, MLN -	Negative	Spl -, PLN -
N202	LF	Ataxia	265	NA	265	NA	Negative	Ton - , PP -, Spl -, PLN -, MLN -	Negative	Spl -
**Negative control sheep**										
N256	LF	Negative	2014	NA	NA	NA	Negative	Ton - , PP -, Spl -	NT	NT
N239	LL	Negative	2014	NA	NA	NA	Negative	Ton - , PP -, Spl -	NT	NT
N173	LF	Negative	2343	NA	NA	NA	Negative	Ton - , PP -, Spl -	NT	NT
N155	FF	Negative	2308	NA	NA	NA	Negative	Ton - , PP -, Spl -	NT	NT
N214	LF	Negative	1588	NA	NA	NA	Negative	Ton - , PP -, Spl -, PLN -, MLN -	Negative	NT
N170	FF	Negative	1785	NA	NA	NA	Negative	Ton - , PP -, Spl -	Negative	PLN -
N217	FF	Negative	1785	NA	NA	NA	Negative	Ton - , PP -, Spl -	Negative	PLN -
N209	FF	Negative	1747	NA	NA	NA	Negative	Ton - , PP -, Spl -, PLN -, MLN -	Negative	PLN -
N152	FF	Negative	1747	NA	NA	NA	Negative	Ton - , PP -, Spl -	Negative	PLN -

Key:		
	dpi	Days post-infection
	%SP	Time of donation x 100/survival period
	NA	Not applicable
	NT	Not tested

Clinical status*	
Positive =	typical clinical signs of BSE
Negative =	healthy at time of euthanasia
Intercurrent =	euthanasia/death due to non-TSE health issue
Ataxia =	idiopathic ataxia

Peripheral tissues**	
Ton	Tonsil
PP	Ileal Peyers patch
Spl	Spleen
PLN	Prescapular lymph node
MLN	Mesenteric lymph node
