# Supplementary material for: Preclinical transmission of prions by blood transfusion is influenced by donor genotype and route of infection
Source: PLoS Pathog. 2021 Feb 18;17(2):e1009276. doi: 10.1371/journal.ppat.1009276 (PMC7891701; doi:10.1371/journal.ppat.1009276)
Supplement: S2 Table — (PDF) [file ppat.1009276.s002.pdf]

S2 Table.

Donor ID	Recipient ID	Component transfused*	*PRNP* codon 141	Clinical status**	Survival period (dpi) - infected recipients	Survival period (dpi) - uninfected recipients	IHC - brain	IHC - peripheral tissues†	Western blot - brain	Western blot - peripheral tissues†
**BSE-infected donor; all recipients infected (5 donors)**										
N257	M246	WB	FF	Positive	567	NA	Positive	Ton +, PP +, Spl +, PLN +. MLN +	Positive	NT
	M455	RCC	FF	Intercurrent	616	NA	Positive	Ton +, PP +, Spl -, PLN -, MLN +	Positive	NT
	N160	BC	FF	Positive	639	NA	Positive	Ton +, PP -, Spl +, PLN +, MLN +	Positive	NT
	M200	PLS	FF	Positive	594	NA	Positive	Ton +, PP +, Spl +, PLN +, MLN +	Positive	Spl +
	N350	PLT	FF	Positive	741	NA	Positive	Ton +, PP +, Spl +, PLN +, MLN +	Positive	NT
N236	N490	RCC	LF	Intercurrent	658	NA	Positive	Ton +, PP +, Spl +, PLN +, MLN -	Positive	Spl +, PLN +
	M519	BC	FF	Positive	904	NA	Positive	Ton +, PP +, Spl +, PLN +, MLN -	Positive	NT
	N348	PLS	LF	Positive	953	NA	Positive	Ton +, PP +, Spl -, PLN +, MLN +	Positive	NT
	M237	PLT	FF	Positive	609	NA	Positive	Ton +, PP +, Spl +, PLN +, MLN +	Positive	NT
	M490	WB	LF	Positive	792	NA	Positive	Ton +, PP +, Spl +, PLN +, MLN +	Positive	NT
N233	N373	RCC-LR	LF	Positive	1008	NA	Positive	Ton +, PP +, Spl -, PLN -, MLN -	Positive	NT
	N313	PLS-LR	FF	Positive	770	NA	Positive	Ton +, PP +, Spl -, PLN +, MLN +	Positive	NT
	N260	PLT-LR	LF	Positive	1120	NA	Positive	Ton +, PP +, Spl +, PLN +, MLN -	Positive	NT
	N542	RCC	LF	Positive	1008	NA	Positive	Ton +, PP +, Spl +, PLN -, MLN -	Positive	NT
	M525	BC	LL	Positive	513	NA	Positive	Ton +, PP +, Spl +, PLN +, MLN +	Positive	NT
	N171	PLS	FF	Positive	720	NA	Positive	Ton +, PP +, Spl +, PLN +, MLN -	Positive	NT
	N192	PLT	LF	Positive	391	NA	Positive	Ton +, PP +, Spl +, PLN -, MLN -	Negative	NT
N251	P553	RCC	LF	Positive	987	NA	Positive	Ton +, PP +, Spl -, PLN -, MLN -	Positive	NT
	P263	BC	FF	Intercurrent	603	NA	Positive	Ton +, PP +, Spl +, PLN +, MLN +	Positive	NT
	P341	PLS	LF	Positive	1089	NA	Positive	Ton +, PP -, Spl +, PLN -, MLN -	Positive	NT
	P210	PLT	LF	Positive	974	NA	Positive	Ton +, PP +, Spl -, PLN -, MLN -	Positive	NT
	P480	WB	LL	Positive	468	NA	Positive	Ton +, PP +, Spl -, PLN -, MLN -	Positive	Spl +
N261	P528	PLS-LR	LF	Positive	1323	NA	Positive	Ton +, PP +, Spl +, PLN -, MLN -	Positive	NT
	P486	PLT-LR	LL	Intercurrent	NA	33	Negative	Ton -, PP -, Spl -, PLN -, MLN -	Negative	PLN -
	P445	BC	LF	Positive	729	NA	Positive	Ton +, PP +, Spl +, PLN +, MLN +	Positive	NT
	P344	PLS	FF	Positive	652	NA	Positive	Ton +, PP +, Spl +, PLN +, MLN +	Positive	NT
	P452	PLT	LF	Positive	1016	NA	Positive	Ton +, PP +, Spl -, PLN -, MLN -	Positive	NT

	P489	RCC-LR	LF	Positive	1245	NA	Positive	Ton +, PP +, Spl +, PLN -, MLN -	Positive	NT
	P526	RCC	LF	Positive	1105	NA	Positive	Ton +, PP +, Spl +, PLN -, MLN +	Positive	NT
**BSE-infected donor; some recipients infected (10 donors)**										
N178	M216	RCC	LL	Intercurrent	NA	1911	Negative	Ton -, PP -, Spl -	Negative	NT
	N224	BC	LL	Intercurrent	631	NA	Positive	Ton +, PP +, Spl +, PLN +, MLN +	Positive	NT
	M524	PLS	LF	Negative	NA	2493	Negative	Ton -, PP -, Spl -, PLN -	NT	Spl -, PP -
	N255	PLT	LF	Negative	NA	2356	Negative	Ton -, PP -, Spl -, PLN -	NT	Spl -, PP -
N245	M241	PLS-LR	LL	Ataxia	NA	2057	Negative	Ton -, Spl -, PLN -	Negative	NT
	N200	PLT-LR	FF	Negative	NA	2183	Negative	Ton -, Spl -, PLN -	Negative	NT
	N325	PLS	FF	Negative	NA	2183	Negative	Ton -, Spl -, PLN -	Negative	NT
	M259	PLT	FF	Positive	1001	NA	Positive	Ton +, PP +, Spl +, PLN +, MLN -	Positive	NT
N189	N244	RCC-LR	LF	Negative	NA	2177	Negative	Ton -, Spl -, PLN -	Negative	NT
	N374	PLS-LR	FF	Negative	NA	2177	Negative	Ton -, Spl -, PLN -	Negative	NT
	M239	PLT-LR	FF	Negative	NA	2179	Negative	Ton -, Spl -, PLN -	Negative	NT
	M546	RCC	FF	Negative	NA	2178	Negative	Ton -, Spl -, PLN -	Negative	NT
	N253	BC	FF	Positive	701	NA	Positive	Ton +, PP +, Spl +, PLN +, MLN +	Positive	NT
	M226	PLS	FF	Negative	NA	2178	Negative	Ton -, Spl -, PLN -	Negative	NT
	N254	PLT	FF	Positive	701	NA	Positive	Ton +, PP +, Spl +, PLN +, MLN +	Positive	NT
N204	M230	RCC-LR	FF	Negative	NA	2043	Negative	Ton -, Spl -, PLN -	Negative	NT
	N176	PLS-LR	FF	Negative	NA	2288	Negative	Ton -, PP -, Spl -, PLN -	NT	Spl -, PP -
	N179	PLT-LR	LL	Negative	NA	917	Negative	Ton -, PP -, Spl -, PLN -, MLN -	Negative	Spl -
	M527	RCC	LL	Negative	NA	2287	Negative	Ton -, PP -, Spl -, PLN -	NT	PP -
	N237	BC	LL	Positive	595	NA	Positive	Ton +, Spl +, PLN +, MLN +	Positive	NT
	M497	PLS	LF	Negative	NA	2288	Negative	Ton -, PP -, Spl -, PLN -	NT	Spl -, PP -
	N369	PLT	FF	Positive	643	NA	Positive	Ton +, PP +, Spl +, PLN +, MLN +	Positive	Spl +
N231	P285	RCC-LR	LF	Negative	NA	2300	Negative	Ton -, PP -, Spl -, PLN -	Negative	Spl -
	P271	PLS-LR	FF	Ataxia	NA	749	Negative	Ton -, PP -, Spl -, PLN -, MLN -	Negative	Spl -
	P222	PLT-LR	NR	Negative	NA	2308	Negative	Ton -, PP -, Spl -, PLN -	NT	Spl -
	P241	RCC	LF	Negative	NA	333	Negative	Ton -, PP -, Spl -, PLN -, MLN -	Negative	Spl -, PLN -
	P226	BC	LF	Positive	1146	NA	Positive	Ton -, PP -, Spl +, PLN -, MLN -	Positive	NT
	P204	PLS	FF	Negative	NA	2308	Negative	Ton -, PP -, Spl -, PLN -	NT	Spl -
	P305	PLT	FF	Negative	NA	2308	Negative	Ton -, PP -, Spl -, PLN -	NT	Spl -
	P490	RCC	LF	Negative	NA	3951	Negative	Ton -, PP -, Spl -, PLN -, MLN -	Negative	Spl -

N218	P542	BC	LF	Negative	NA	3829	Negative	Ton -, PP -, Spl -, PLN -, MLN -	Negative	Spl -
	P231	PLS	LF	Ataxia	NA	1519	Negative	Ton -, PP -, Spl -	Negative	NT
	P561	PLT	LF	Negative	NA	3948	Negative	Ton -, PP -, Spl -, PLN -, MLN -	Negative	Spl -
	P453	WB	LF	Positive	1199	NA	Positive	Ton -, PP +, Spl +, PLN -, MLN +	Positive	NT
N232	P213	RCC-LR	LF	Positive	681	NA	Positive	Ton +, PP +, Spl +, PLN +, MLN +	Positive	Spl +
	P429	PLS-LR	LF	Negative	NA	4000	Negative	Ton -, PP -, Spl -, PLN -, MLN -	Negative	Spl -
	P463	PLT-LR	FF	Negative	NA	2936	Negative	Ton -, PP -, Spl -	Negative	Spl -
	P482	BC	LF	Positive	878	NA	Positive	Ton +, PP +, Spl +, PLN +, MLN -	Positive	NT
	P290	PLS	LF	Positive	1234	NA	Positive	Ton +, PP +, Spl +, PLN -, MLN -	Positive	NT
	P547	PLT	LF	Positive	995	NA	Positive	Ton +, PP +, Spl +, PLN -, MLN -	Positive	NT
	P536	RCC	FF	Positive	841	NA	Positive	Ton +, PP +, Spl -, PLN +, MLN +	Positive	NT
N226	P477	PLS-LR	LF	Negative	NA	3946	Negative	Ton -, PP -, Spl -, PLN -, MLN -	Negative	Spl -
	P521	PLT-LR	LF	Ataxia	NA	1826	Negative	Ton -, Spl -, PLN -	Negative	NT
	P224	BC	LF	Positive	784	NA	Positive	Ton +, PP +, Spl +, PLN -, MLN -	Positive	NT
	P525	PLS	LF	Intercurrent	NA	2163	Negative	Ton -, PP -, Spl -, PLN -	NT	Spl -, PP -
	P483	PLT	LF	Negative	NA	3961	Negative	Ton -, PP -, Spl -, PLN -, MLN -	Negative	Spl -
	P431	RCC-LR	LF	Intercurrent	NA	3428	Negative	Ton -, PP -, Spl -, PLN -, MLN -	Negative	Spl -
	P428	RCC	LF	Intercurrent	NA	2800	Negative	Ton -, Spl -, PLN -	Negative	Spl -
N157	P485	PLS-LR	FF	Negative	NA	3940	Negative	Ton -, PP -, Spl -, PLN -, MLN -	Negative	Spl -
	P432	PLT-LR	FF	Intercurrent	NA	2520	Positive (AS)	PP -, Spl -, PLN -	Positive (AS)	Spl -
	P238	BC	FF	Positive	821	NA	Positive	Ton +, PP +, Spl +, PLN +, MLN -	Positive	NT
	P456	PLS	LF	Negative	NA	3529	Negative	Ton -, PP -, Spl -, PLN -, MLN -	Negative	Spl -
	P515	PLT	FF	Intercurrent	NA	3898	Negative	Ton -, PP -, Spl -, PLN -, MLN -	Negative	Spl -
	P496	RCC-LR	FF	Intercurrent	NA	1614	Positive (AS)	Ton -, PP -, Spl -	Positive (AS)	NT
	P527	RCC	LF	Positive	1063	NA	Positive	Ton +, PP +, Spl +, PLN +, MLN +	Positive	NT
N180	P427	RCC-LR	LF	Intercurrent	NA	2631	Negative	Ton -, Spl -, PLN -	Negative	Spl -
	P488	PLS-LR	FF	Intercurrent	NA	3250	NT	NT	Negative	Spl -
	P342	PLT-LR	FF	Intercurrent	NA	1983	Negative	Ton -, Spl -, PLN -	Negative	NT
	P451	RCC	LF	Intercurrent	1309	NA	Positive	Ton -, PP +, Spl +, PLN +, MLN -	Positive	NT
	P228	BC	LF	Intercurrent	1199	NA	Positive	Ton -, PP -, Spl -, PLN -, MLN -	Positive	NT
	P545	PLS	LF	Negative	NA	3982	Negative	Ton -, PP -, Spl -, PLN -, MLN -	Negative	Spl -
	P460	PLT	LF	Positive	1135	NA	Positive	Ton +, PP +, Spl +, PLN -, MLN -	Positive	NT
**BSE-infected donor; no recipients infected (18 donors)**										

N196	M172	RCC-LR	FF	Negative	NA	2135	Negative	Ton -, Spl -, PLN -	Negative	NT
	M257	PLS-LR	LF	Negative	NA	2135	Negative	Ton -, Spl -, PLN -	Negative	NT
	M520	PLT-LR	FF	Negative	NA	2136	Negative	Ton -, Spl -, PLN -	Negative	NT
	N221	RCC	LF	Negative	NA	2107	Negative	Ton -, Spl -, PLN -	Negative	NT
	N213	BC	LF	Ataxia	NA	73	Negative	Ton -, PP -, Spl -, PLN -, MLN -	Negative	NT
	N481	PLS	FF	Negative	NA	2136	Negative	Ton -, Spl -, PLN -	Negative	NT
N206	N252	RCC-LR	LL	Negative	NA	2134	Negative	Ton -, Spl -, PLN -	Negative	NT
	M305	BC	FF	Negative	NA	2134	Negative	Ton -, Spl -, PLN -	Negative	NT
	N211	PLS-LR	FF	Negative	NA	2134	Negative	Spl -, PLN -	Negative	NT
	M556	PLT-LR	FF	Negative	NA	2135	Negative	Ton -, Spl -, PLN -	Negative	NT
	N547	RCC	FF	Negative	NA	2161	Negative	Ton -, Spl -, PLN -	Negative	NT
	N156	PLS	FF	Negative	NA	2161	Negative	Ton -, Spl -, PLN -	Negative	NT
	M165	PLT	LF	Negative	NA	2107	Negative	Ton -, Spl -, PLN -	Negative	NT
N264	M220	RCC-LR	FF	Negative	NA	2156	Negative	Ton -, Spl -, PLN -	Negative	NT
	N344	PLS-LR	FF	Negative	NA	2156	Negative	Ton -, Spl -, PLN -	Negative	NT
	N153	PLT-LR	LF	Intercurrent	NA	899	Negative	Ton -, PP -, Spl -, PLN -, MLN -	Negative	Spl -
	N363	RCC	LL	Negative	NA	2157	Negative	Ton -, Spl -, PLN -	Negative	NT
	M532	BC	LF	Negative	NA	2158	Negative	Ton -, Spl -, PLN -	Negative	NT
	N349	PLS	LF	Negative	NA	2157	Negative	Ton -, Spl -, PLN -	Negative	NT
	M271	PLT	LF	Ataxia	NA	590	Negative	Ton -, PP -, Spl -, PLN -, MLN -	Negative	Spl -
N175	M179	RCC-LR	LF	Intercurrent	NA	694	Negative	PP -, Spl -, PLN -, MLN -	Negative	Spl -
	N480	BC	LF	Negative	NA	2177	Negative	Spl -, PLN -	Negative	NT
	N185	PLS-LR	LF	Negative	NA	2177	Negative	Ton -, Spl -, PLN -	Negative	NT
	N163	PLT-LR	LL	Intercurrent	NA	806	Negative	Ton -, PP -, Spl -, PLN -, MLN -	Negative	PLN -
N246	N172	RCC-LR	LF	Negative	NA	2253	Negative	Ton -, PP -, Spl -	NT	Spl - PP -
	M252	PLS-LR	LF	Negative	NA	1903	Negative	Ton -, PP -, Spl -	Negative	NT
	N195	PLT-LR	LF	Negative	NA	2254	Negative	Ton -, PP -, Spl -	NT	Spl - PP -
	M502	RCC	LF	Negative	NA	2253	Negative	Ton -, PP -, Spl -, PLN -	NT	Spl - PP -
	N519	BC	LF	Negative	NA	2254	Negative	Ton -, PP -, Spl -	NT	Spl - PP -
	M561	PLS	FF	Positive	NA	1681	Negative	Ton -, PP -, Spl -	Negative	NT
	N235	PLT	LF	Intercurrent	NA	1674	Negative	Ton -, PP -, Spl -, PLN -, MLN -	Negative	NT
	M227	RCC-LR	LF	Ataxia	NA	1751	Negative	Ton -, PP -, Spl -, PLN -	Negative	NT
	N212	PLS-LR	LF	Ataxia	NA	946	Negative	Ton -, PP -, Spl -, PLN -, MLN -	Negative	Spl -

N241	N225	PLT-LR	FF	Negative	NA	2249	Negative	Ton -, PP -, Spl -	NT	Spl - PP -
	N207	RCC	FF	Intercurrent	NA	1724	Negative	Ton -, PP -, Spl -	Negative	NT
	M522	BC	LF	Negative	NA	2277	Negative	Ton -, PP -, Spl -	NT	Spl - PP -
	N197	PLS	LL	Negative	NA	2276	Negative	Ton -, PP -, Spl -	NT	Spl - PP -
	M505	PLT	FF	Intercurrent	NA	1472	Positive (AS)	Ton -, PP -, Spl -, PLN -, MLN -	Positive (AS)	PLN -
N234	N459	RCC	LF	Ataxia	NA	1245	Negative	Ton -, PP -, Spl -, PLN -, MLN -	Negative	NT
	M260	BC	FF	Intercurrent	NA	981	Positive (AS)	Ton -, PP -, Spl -, PLN -, MLN -	Positive (AS)	PLN -
	N370	PLS	FF	Negative	NA	2259	NT	NT	NT	Spl - PP -
	M360	PLT	LF	Positive	NA	2049	Negative	PP -, Spl -	Negative	NT
	M250	WB	FF	Negative	NA	2259	Negative	Ton -, PP -, Spl -	NT	Spl - PP -
N223	N190	RCC-LR	LF	Negative	NA	2248	Negative	Ton -, PP -, Spl -, PLN -	NT	Spl - PP -
	N347	PLS-LR	LF	Negative	NA	2249	Negative	Ton -	NT	Spl - PP -
	N242	PLT-LR	LL	Negative	NA	2249	Negative	Ton -, PP -, Spl -, PLN -	NT	Spl - PP -
	N314	RCC	LF	Negative	NA	2248	Negative	Ton -, PP -, Spl -, PLN -	NT	Spl - PP -
	M177	BC	FF	Negative	NA	2248	Negative	Ton -, PP -, Spl -	NT	Spl - PP -
	N266	PLS	FF	Ataxia/weight loss	NA	1404	Positive (AS)	Ton -, PP -, Spl -, PLN -, MLN -	Positive (AS)	PLN -
	N326	PLT	FF	Negative	NA	2283	Positive (AS)	Ton -, PP -, Spl -, PLN -	Positive (AS)	Spl - PP -
N258	N355	RCC-LR	LL	Negative	NA	2280	Negative	Ton -, PP -, Spl -, PLN -	NT	Spl - PP -
	M452	PLS-LR	FF	Intercurrent	NA	2247	Negative	Ton -, PP -, Spl -, PLN -	NT	Spl - PP -
	N483	PLT-LR	LF	Negative	NA	2282	Negative	Ton -, PP -, Spl -, PLN -	NT	Spl - PP -
	N174	RCC	LL	Negative	NA	2281	Negative	Ton -, PP -, Spl -, PLN -	NT	Spl - PP -
	M518	BC	FF	Positive	NA	603	Negative	Ton -, PP -, Spl -, PLN -, MLN -	Negative	Spl -
	N249	PLS	LF	Ataxia	NA	391	Negative	Ton -, PP -, Spl -, PLN -, MLN -	Negative	Spl -, PLN -
	N387	PLT	LF	Negative	NA	2444	Negative	Ton -, PP -, Spl -, PLN -	NT	Spl - PP -
N220	N383	RCC-LR	FF	Intercurrent	NA	1920	Negative	Spl -, PLN -	Negative	NT
	N437	RCC	LF	Intercurrent	NA	2003	Negative	Ton -, Spl -, PLN -	Negative	NT
	M538	BC	LL	Ataxia	NA	400	Negative	Ton -, PP -, Spl -, PLN -, MLN -	Negative	Spl -
	N359	PLS	LF	Negative	NA	2031	Negative	Ton -, Spl -, PLN -	Negative	NT
	N243	PLT	LF	Negative	NA	2324	Negative	Ton -, Spl -, PLN -	NT	Spl -
N188	P220	RCC	FF	Negative	NA	2324	Negative	Ton -, PP -, Spl -, PLN -	NT	Spl -
	P243	BC	LF	Negative	NA	2323	Negative	Ton -, PP -, Spl -, PLN -	NT	Spl -
	P237	PLS	FF	Negative	NA	2324	Negative	Ton -, PP -, Spl -, PLN -	NT	Spl -
	P279	PLT	FF	Ataxia	NA	1944	Negative	Ton -, Spl -, PLN -	Negative	NT

	P177	WB	LF	Negative	NA	2218	Negative	Ton -, PP -, Spl -	NT	Spl - PP -
N161	P244	RCC-LR	LF	Ataxia	NA	142	Negative	Ton -, PP -, Spl -, PLN -, MLN -	Negative	Spl -
	P289	PLS-LR	FF	Negative	NA	2304	Negative	Ton -, PP -, Spl -, PLN -	Negative	Spl -
	P334	PLT-LR	FF	Negative	NA	2304	Negative	PP -, Spl -, PLN -	Negative	Spl -
	P306	RCC	LF	Ataxia	NA	1603	Negative	Ton -, PP -, Spl -	Negative	NT
	P208	BC	FF	Negative	NA	2304	Negative	Ton -, PP -, Spl -, PLN -	Negative	Spl -
	P227	PLS	FF	Intercurrent	NA	414	Negative	Ton -, PP -, Spl -, PLN -, MLN -	Negative	Spl -
	P264	PLT	LL	Negative	NA	2304	Negative	Ton -, PP -, Spl -, PLN -	Negative	Spl -
N228	P458	RCC / BC MIX	LF	Ataxia	NA	1634	Negative	Ton -, PP -, Spl -	Negative	NT
	P506	PLS	FF	Negative	NA	2265	Negative	Ton -, PP -, Spl -, PLN -	NT	Spl - PP -
	P343	PLT	FF	Positive	NA	788	Negative	Ton -, PP -, Spl -, PLN -, MLN -	Negative	NT
	P248	WB	LF	Intercurrent	NA	2102	Negative	Ton -, PP -, Spl -, PLN -	NT	Spl - PP -
N164	P307	RCC-LR	LF	Intercurrent	NA	1008	Negative	Ton -, PP -, Spl -, PLN -, MLN -	Negative	NT
	P292	PLS-LR	LF	Intercurrent	NA	1390	Negative	Ton -, PP -, Spl -, PLN -, MLN -	Negative	NT
	P340	PLT-LR	LF	Negative	NA	4014	Negative	Ton -, PP -, Spl -, PLN -, MLN -	Negative	Spl -
	P245	RCC	FF	Weight loss	NA	1406	Positive (AS)	Ton -, PP -, Spl -, PLN -, MLN -	Positive (AS)	NT
	P230	BC	FF	Negative	NA	1968	Negative	Ton -, PP -, Spl -	Negative	NT
	P214	PLS	LL	Intercurrent	NA	2517	Negative	Ton -, PP -, Spl -, PLN -	Negative	Spl -
	P273	PLT	FF	Intercurrent	NA	3278	NT	NT	Negative	Spl -
N208	P309	RCC-LR	FF	Ataxia	NA	1966	Negative	Ton -, PP -, Spl -	Negative	NT
	P294	PLS-LR	FF	Ataxia	NA	588	Negative	Ton -, Spl -, PLN -, MLN -	Negative	Spl -
	P347	PLT-LR	LL	Negative	NA	3890	Negative	Ton -, PP -, Spl -, PLN -, MLN -	Negative	Spl -
	P254	RCC	LF	Negative	NA	4015	Negative	Ton -, PP -, Spl -, PLN -, MLN -	Negative	Spl -
	P217	BC	LF	Intercurrent	NA	3138	Negative	Ton -, PP -, Spl -	Negative	Spl -
	P351	PLS	LL	Negative	NA	4009	Negative	Ton -, PP -, Spl -, PLN -, MLN -	Negative	Spl -
	P275	PLT	LF	Ataxia	NA	587	Negative	Ton -,PP -, Spl -, PLN -, MLN -	Negative	Spl -
N248	P229	RCC-LR	LF	Intercurrent	NA	3269	NT	NT	Negative	Spl -
	P563	PLS-LR	LF	Intercurrent	NA	3580	Negative	Ton -, PP -, Spl -, PLN -, MLN -	Negative	Spl -
	P196	PLT-LR	LF	Intercurrent	NA	1977	Negative	Ton -, PP -, Spl -	Negative	NT
	N544	RCC	LF	Intercurrent	NA	2663	NT	NT	Negative	Spl -
	P464	BC	LF	Ataxia	NA	2550	Positive (AS)	Ton -, PP -, Spl -, PLN -	Positive (AS)	Spl -
	P261	PLS	LL	Negative	NA	3982	Negative	Ton -, PP -, Spl -, PLN -, MLN -	Negative	Spl -
	P409	PLT	FF	Intercurrent	NA	3272	NT	NT	Negative	Spl -

N259	P493	RCC	FF	Positive	NA	2138	Negative	Ton -, PP -, Spl -, PLN -	NT	Spl -, PP -
	P353	BC	LF	Intercurrent	NA	2109	Negative	Ton -, PP -, Spl -	NT	Spl -, PP -
	P236	PLS	FF	Intercurrent	NA	3549	Negative	Ton -, PP -, Spl -, PLN -, MLN -	NT	NT
	P422	PLT	LF	Negative	NA	3958	Negative	Ton -, PP -, Spl -, PLN -, MLN -	Negative	Spl -
	P455	WB	LF	Negative	NA	3955	Negative	Ton -, PP -, Spl -, PLN -, MLN -	Negative	Spl -
N169	P531	PLS-LR	FF	Ataxia	NA	790	Negative	Ton -, PP -, Spl -, PLN -, MLN -	Negative	Spl -
	P442	PLT-LR	LF	Intercurrent	NA	2191	Negative	Ton -, PP -, Spl -, PLN -	Negative	Spl -, PP -
	P225	BC	LF	Ataxia	NA	503	Negative	Ton -, PP -, Spl -, PLN -, MLN -	Negative	Spl -
	P550	PLS	LF	Intercurrent	NA	961	Negative	Ton -, PP -, Spl -, PLN -, MLN -	Negative	Spl -, PP -
	P522	PLT	LF	Ataxia	NA	1791	Negative	Ton -, PP -, Spl -	Negative	NT
	P324	RCC-LR	LF	Negative	NA	2353	Negative	Ton -, PP -, Spl -, PLN -	Negative	Spl -, PP -
	P484	RCC	LF	Negative	NA	2353	Negative	Ton -, PP -, Spl -, PLN -	Negative	Spl -, PP -
**Infection not confirmed in donor; no infected recipients (6 donors)**										
N201	N457	RCC-LR	LF	Negative	NA	2191	Negative	Ton -, Spl -, PLN -	Negative	NT
	N482	PLS-LR	LF	Negative	NA	2191	Negative	Ton -, Spl -, PLN -	Negative	NT
	M253	PLT-LR	LL	Negative	NA	2193	Negative	Ton -, Spl -, PLN -	Negative	NT
	M215	RCC	LF	Negative	NA	2191	Negative	Ton -, Spl -, PLN -	Negative	NT
	N375	BC	LF	Negative	NA	2192	Negative	Ton -, Spl -, PLN -	Negative	NT
	M356	PLS	LF	Negative	NA	2192	Negative	Ton -, Spl -, PLN -	Negative	NT
	N210	PLT	FF	Negative	NA	2193	Negative	Ton -, Spl -, PLN -	Negative	NT
N159	N167	RCC-LR	LF	Negative	NA	2245	Negative	Ton -, PP -, Spl -, PLN -	Negative	Spl -, PP -
	M258	PLS-LR	LF	Negative	NA	2065	Negative	Ton -, Spl -, PLN -	Negative	NT
	N343	PLT-LR	LL	Negative	NA	2246	Negative	Ton -, Spl -, PLN -	Negative	Spl -, PP -
	N312	RCC	LF	Intercurrent	NA	15	Negative	Ton -, PP -, Spl -, PLN -, MLN -	Negative	NT
	M499	BC	FF	Negative	NA	2246	Negative	Ton -, PP -, Spl -, PLN -	Negative	Spl -, PP -
	N333	PLS	LF	Negative	NA	2246	Negative	Ton -, PP -, Spl -, PLN -	Negative	Spl -, PP -
	M254	PLT	FF	Intercurrent	NA	2274	Negative	Ton -, PP -, Spl -	Negative	Spl -, PP -
N181	N247	RCC-LR	LF	Negative	NA	2164	Negative	Ton -, Spl -, PLN -	Negative	NT
	N436	PLS-LR	LF	Intercurrent	NA	1436	Negative	Ton -, PP -, Spl -, PLN -, MLN -	Negative	NT
	M228	PLT-LR	LF	Intercurrent	NA	1920	Negative	Ton -, Spl -, PLN -	Negative	NT
	M466	RCC	LF	Intercurrent	NA	688	Negative	Ton -, PP -, Spl -, PLN -, MLN -	Negative	Spl -
	N388	BC	FF	Negative	NA	2165	Negative	Ton -, Spl -, PLN -	Negative	NT
	M521	PLS	LF	Negative	NA	2165	Negative	Ton -, Spl -, PLN -	Negative	NT

	N194	PLT	LF	Negative	NA	2227	Negative	PP -, Spl -, PLN -	Negative	Spl -, PP -
N219	P300	RCC-LR	LF	Negative	NA	2309	Negative	Ton -, PP -, Spl -, PLN -	NT	Spl -
	P284	PLS-LR	LF	Intercurrent	NA	2040	Negative	Ton -, Spl -, PLN -	Negative	NT
	P313	PLT-LR	FF	Negative	NA	2304	Negative	Ton -, PP -, Spl -, PLN -	NT	Spl -
	P240	RCC	FF	Negative	NA	2303	Negative	Ton -, PP -, Spl -, PLN -	NT	Spl -
	P180	BC	LF	Negative	NA	2304	Negative	Ton -, PP -, Spl -, PLN -	NT	Spl -
	P221	PLS	LF	Intercurrent	NA	1618	Negative	Ton -, PP -, Spl -, PLN -, MLN -	Negative	NT
	P268	PLT	LL	Negative	NA	2304	Negative	Ton -, PP -, Spl -, PLN -	NT	Spl -
N250	P311	RCC-LR	LF	Negative	NA	2106	Negative	Ton -, Spl -, PLN -	Negative	NT
	P297	PLS-LR	FF	Negative	NA	2303	Negative	PP -, Spl -	NT	Spl -
	P350	PLT-LR	LF	Ataxia	NA	1780	Negative	Ton -, PP -, Spl -	Negative	NT
	P258	RCC	FF	Ataxia	NA	1986	Negative	Ton -, Spl -, PLN -	Negative	NT
	P219	BC	LL	Negative	NA	2303	Negative	Ton -, PP -, Spl -, PLN -	NT	Spl -
	P235	PLS	FF	Negative	NA	2303	Negative	Ton -, PP -, Spl -, PLN -, MLN -	NT	Spl -
	P278	PLT	FF	Negative	NA	2393	Negative	Ton -, PP -, Spl -, PLN -	NT	Spl -, PP -
N202	P462	RCC-LR	LF	Negative	NA	2385	Negative	Ton -, PP -, Spl -, PLN -	NT	Spl -, PP -
	P537	PLS-LR	LF	Negative	NA	2386	Negative	Ton -, PP -, Spl -, PLN -	NT	Spl -, PP -
	P441	PLT-LR	LF	Negative	NA	2388	Negative	Ton -, Spl -, PLN -	NT	Spl -, PP -
	P413	RCC	FF	Intercurrent	NA	1273	Negative	Ton -, PP -, Spl -, PLN -, MLN -	Negative	NT
	M515	BC	FF	Intercurrent	NA	1855	Negative	Ton -, PP -, Spl -, PLN -	Negative	NT
	P251	PLS	LL	Positive	NA	1557	Negative	Ton -, PP -, Spl -, PLN -, MLN -	Negative	NT
	P187	PLT	LL	Negative	NA	1981	Negative	Spl -, PLN -	Negative	NT
**Negative control sheep**										
N256	M278	WB	FF	Weight loss	NA	1114	Positive (AS)	Ton -, PP -, Spl -	Positive (AS)	PLN -
N239	M308	WB	LL	Negative	NA	2142	Negative	Ton -, PP -, Spl -, PLN -, MLN -	Negative	NT
N173	M481	WB	LF	Intercurrent	NA	2843	Negative	Ton -, Spl -, PLN -	Negative	Spl -
N155	P205	WB	LL	Ataxia	NA	774	Negative	Ton -, PP -, Spl -, PLN -, MLN -	Negative	PLN -
N214	P260	WB	LF	Intercurrent	NA	3434	Negative	Ton -, PP -, Spl -, PLN -, MLN -	Negative	Spl -
N170	P194	WB	FF	Intercurrent	NA	1364	Negative	Ton -, PP -, Spl -, PLN -, MLN -	Negative	NT
N217	P423	WB	LF	Intercurrent	NA	312	Negative	Ton -, Spl -, PLN -, MLN -	Negative	Spl -, PLN -
N209	P457	WB	LF	Intercurrent	NA	2402	Negative	Ton -, PP -, Spl -, PLN -	Negative	Spl -
N152	P242	WB	LF	Negative	NA	3944	Negative	Ton -, PP -, Spl -, PLN -, MLN -	Negative	Spl -

Key:		
	dpi	Days post-infection
	NA	Not applicable
	NT	Not tested
	AS	Atypical scrapie

Component transfused*

WB	Whole blood
BC	Buffy coat
RCC	Red cell concentrate
PLT	Platelets
PLS	Plasma
RCC-LR	Leucodepleted red cell concentrate
PLT-LR	Leucodepleted platelets
PLS-LR	Leucodepleted plasma

Peripheral tissues†

Ton	Tonsil
PP	Ileal Peyers patch
Spl	Spleen
PLN	Prescapular lymph node
MLN	Mesenteric lymph node

Clinical status**

Positive =	typical clinical signs of BSE
Negative =	healthy at time of euthanasia
Intercurrent =	euthanasia/death due to non-TSE health issue
Ataxia =	idiopathic ataxia
