# Supplementary material for: Preclinical transmission of prions by blood transfusion is influenced by donor genotype and route of infection
Source: PLoS Pathog. 2021 Feb 18;17(2):e1009276. doi: 10.1371/journal.ppat.1009276 (PMC7891701; doi:10.1371/journal.ppat.1009276)
Supplement: S3 Table — The inoculum was prepared from a single sheep infected with BSE by intracerebral inoculation of BSE-infected cattle brain homogenate. Each mouse was inoculated intracerebrally under general anaesthesia with 25 μl of the brain dilution indicated, and monitored for development of clinical signs up to 700 days post infection. Brains from all mice were tested for the presence of PrPSc using an ELISA kit (BetaPrion BSE EIA Test Kit; AJ Roboscreen Gmbh, Leipzig, Germany) to confirm infection status. (DOCX) [file ppat.1009276.s003.docx]

**Table S3**

| **Log_10_ brain dilution** | **Number of mice inoculated** | **Number of mice infected (BSE positive)** |
| --- | --- | --- |
| -1 | 10 | 10 |
| -2 | 11 | 11 |
| -3 | 9 | 9 |
| -4 | 9 | 9 |
| -5 | 10 | 10 |
| -6 | 10 | 5 |
| -7 | 10 | 1 |
| -8 | 10 | 0 |
|  |  |  |
