# Supplementary material for: Preclinical transmission of prions by blood transfusion is influenced by donor genotype and route of infection
Source: PLoS Pathog. 2021 Feb 18;17(2):e1009276. doi: 10.1371/journal.ppat.1009276 (PMC7891701; doi:10.1371/journal.ppat.1009276)
Supplement: S4 Table — (DOCX) [file ppat.1009276.s004.docx]

**S4 Table**

| **Primary recipients (donors)** | | | | | | **Secondary recipients** | | | | | |
| --- | --- | --- | --- | --- | --- | --- | --- | --- | --- | --- | --- |
| **Sheep ID** | ***PRNP* codon 141** | **Survival period – infected (dpi)** | **Survival period – uninfected (dpi)** | **Clinical status^a^** | **IHC/Western blot results on brain & lymphoid tissues^b^** | **Sheep ID** | ***PRNP* codon 141** | **Survival period - infected (dpi)** | **Survival period -uninfected (dpi)** | **Clinical status^a^** | **IHC/Western blot results on brain & lymphoid tissues^b^** |
| N224 | LL | 631 | - | Intercurrent | Positive | P301 | LF | 924 | - | Positive | Positive |
| P480 | LL | 468 | - | Positive | Positive | P508 | LF | 1179 | - | Positive | Positive |
| N160 | FF | 639 | - | Positive | Positive | Q283 | LF | 959 | - | Positive | Positive |
| M246 | FF | 567 | - | Positive | Positive | Q213 | LF | 909 | - | Positive | Positive |
| M519 | FF | 904 | - | Positive | Positive | Q219 | LF | 902 | - | Positive | Positive |
| P263 | FF | 603 | - | Intercurrent | Positive | P502 | FF | 624 | - | Positive | Positive |
| M490 | LF | 792 | - | Positive | Positive | P499 | LF | 940 | - | Positive | Positive |
| P453 | LF | 1199 | - | Positive | Positive | Q273 | LF | 930 | - | Positive | Positive |
| M250 | FF | - | 2259 | Negative | Negative | P308 | LF | - | 1911 | Ataxia | Negative |
| M260 | FF | - | 981 | Intercurrent | Positive (AS)^c^ | Q225 | LF | - | 3461 | Negative | Negative |
| M177 | FF | - | 2248 | Negative | Negative | Q387 | LL | - | 3455 | Negative | Negative |
| P243 | LF | - | 2323 | Negative | Negative | P500 | LF | - | 3384 | Intercurrent | Negative |
| P177 | LF | - | 2218 | Negative | Negative | Q228 | LF | - | 1858 | Ataxia | Negative |
| P353 | LF | - | 2109 | Intercurrent | Negative | Q380 | LF | - | 1785 | Ataxia | Negative |
| P455 | LF | - | 3955 | Negative | Negative | P538 | LF | - | 3463 | Negative | Negative |
| P542 | LF | - | 3829 | Negative | Negative | Q236 | LF | - | 3455 | Negative | Negative |
| P458 | LF | - | 1634 | Ataxia | Negative | Q274 | LF | - | 3254 | Intercurrent | Negative |
| P248 | LF | - | 2102 | Intercurrent | Negative | Q282 | LF | - | 1718 | Ataxia | Negative |

Key: dpi = days post infection

a – Positive = typical clinical signs for BSE; negative = healthy at time of euthanasia; ataxia = idiopathic ataxia; intercurrent = euthanasia/death due to non-TSE health issue

b - details of the Western blot and IHC data for each sheep are in Table S7.

c – this sheep was diagnosed as having atypical scrapie (AS). Atypical scrapie can be clearly distinguished from BSE, because vacuolation and PrP^Sc^ deposition are found predominantly in the cerebral cortex and cerebellum, rather than the brainstem, Western blots show a distinctive 10-12 kDa band, and PrP^Sc^ cannot be detected in lymphoid tissues by IHC/Western blot.
