# Supplementary material for: Preclinical transmission of prions by blood transfusion is influenced by donor genotype and route of infection
Source: PLoS Pathog. 2021 Feb 18;17(2):e1009276. doi: 10.1371/journal.ppat.1009276 (PMC7891701; doi:10.1371/journal.ppat.1009276)
Supplement: S5 Table — (PDF) [file ppat.1009276.s005.pdf]

S5 Table.

Donor ID	Recipient ID	*PRNP* codon 141	Clinical status*	Survival period (dpi) - infected recipients	Survival period (dpi) - uninfected recipients	IHC - brain	IHC - peripheral tissues**	Western blot - brain	Western blot - peripheral tissues**
N224	P301	LF	Positive	924	-	Positive	Ton -, PP +, Spl +, PLN +, MLN -	Positive	NT
P480	P508	LF	Positive	1179	-	Positive	Ton -, PP -, Spl -, PLN +, MLN -	Positive	NT
N160	Q283	LF	Positive	959	-	Positive	Ton -, PP +, Spl +, PLN +, MLN -	Positive	NT
M246	Q213	LF	Positive	909	-	Positive	Ton -, PP +, Spl +, PLN -, MLN -	Positive	NT
M519	Q219	LF	Positive	902	-	Positive	Ton +, PP +, Spl +, PLN -, MLN -	Positive	NT
P263	P502	FF	Positive	624	-	Positive	Ton +, PP +, Spl +, PLN +, MLN -	Positive	NT
M490	P499	LF	Positive	940	-	Positive	Ton -, PP +, Spl +, PLN +, MLN -	Positive	NT
P453	Q273	LF	Positive	930	-	Positive	Ton +, PP +, Spl +, PLN -, MLN -	Positive	NT
M250	P308	LF	Ataxia	-	1911	Negative	Ton -, PP -, Spl -, PLN -, MLN -	NT	Spl -
M260	Q225	LF	Negative	-	3461	Negative	Ton -, PP -, Spl -, PLN -, MLN -	Negative	Spl -
M177	Q387	LL	Negative	-	3455	Negative	Ton -, PP -, Spl -, PLN -, MLN -	Negative	Spl -
P243	P500	LF	Intercurrent	-	3384	Negative	Ton -, PP -, Spl -, PLN -, MLN -	Negative	Spl -
P177	Q228	LF	Ataxia	-	1858	Negative	Ton -, PP -, Spl -, PLN -, MLN -	NT	Spl -, PP -
P353	Q380	LF	Ataxia	-	1785	Negative	Ton -, PP -, Spl -, PLN -, MLN -	NT	Spl -, PP -
P455	P538	LF	Negative	-	3463	Negative	Ton -, PP -, Spl -, PLN -, MLN -	Negative	Spl -
P542	Q236	LF	Negative	-	3455	Negative	Ton -, PP -, Spl -, PLN -, MLN -	Negative	Spl -
P458	Q274	LF	Intercurrent	-	3254	Negative	Ton -, PP -, Spl -, PLN -, MLN -	Negative	Spl -
P248	Q282	LF	Ataxia	-	1718	Negative	Ton -, PP -, Spl -, PLN -, MLN -	NT	Spl -, PP -

Key:
dpi Days post-infection
NT Not tested

Clinical status*
Positive = typical clinical signs of BSE
Negative = healthy at time of euthanasia
Intercurrent = euthanasia/death due to non-TSE health issue
Ataxia = idiopathic ataxia

Peripheral tissues**
Ton Tonsil
PP Ileal Peyers patch
Spl Spleen
PLN Prescapular lymph node
MLN Mesenteric lymph node
