# Supplementary material for: Preclinical transmission of prions by blood transfusion is influenced by donor genotype and route of infection
Source: PLoS Pathog. 2021 Feb 18;17(2):e1009276. doi: 10.1371/journal.ppat.1009276 (PMC7891701; doi:10.1371/journal.ppat.1009276)
Supplement: S6 Table — Each mouse was inoculated intracerebrally under general anaesthesia with 25μl of the indicated components, diluted 1:1 in sterile PBS (dilution was necessary due to acute toxicity associated with inoculation of blood components collected with EDTA as anticoagulant), and monitored for development of clinical signs up to 700 days post infection. Brains from all mice were tested for the presence of PrPSc using an ELISA kit (BetaPrion BSE EIA Test Kit; AJ Roboscreen Gmbh, Leipzig, Germany) to confirm infection status. There were no transmissions in groups of mice injected with samples from time points 0, 4 and 8 months post-infection (data not shown). Infectious titres (ID/ml) were calculated by limiting dilution titration (where distribution of infectivity into individual inoculations is assumed to follow a Poisson distribution). NA–not applicable. (DOCX) [file ppat.1009276.s006.docx]

**S6 Table**

| **Donor sheep ID** | **Blood component** | **Time point** | | | | | |
| --- | --- | --- | --- | --- | --- | --- | --- |
|  |  | **12 mpi** | | **18 mpi** | | **Terminal (18-20 mpi)** | |
|  |  | **No. positive mice/ no. injected** | **Titre (ID/ml)** | **No. positive mice/ no. injected** | **Titre (ID/ml)** | **No. positive mice/ no. injected** | **Titre (ID/ml)** |
| N196 | Whole blood | 1/81 | 1.0 | 0/83 | - | 2/80 | 2.0 |
|  | Buffy coat | 0/32 | - | 0/31 | - | 1/31 | 2.6 |
|  | Plasma | 0/25 | - | 0/25 | - | 0/26 | - |
|  | Red cells | 0/26 | - | 0/27 | - | 0/26 | - |
| N231 | Whole blood | 0/91 | - | 0/89 | - | 3/89 | 2.7 |
|  | Plasma | 0/27 | - | 0/27 | - | 0/27 | - |
|  | Red cells | 0/27 | - | 0/27 | - | 0/27 | - |
| N251 | Whole blood | 0/88 | - | NA | NA | 2/90 | 1.8 |
| N257 | Whole blood | 0/90 | - | 0/90 | - | 0/90 | - |
