# Supplementary material for: Preclinical transmission of prions by blood transfusion is influenced by donor genotype and route of infection
Source: PLoS Pathog. 2021 Feb 18;17(2):e1009276. doi: 10.1371/journal.ppat.1009276 (PMC7891701; doi:10.1371/journal.ppat.1009276)
Supplement: S7 Table — Samples of buffy coat and plasma (undiluted and diluted 1:1 with PMCA buffer) from 4 donor and 10 recipient sheep clinically affected with BSE and 4 negative controls were used to seed PMCA reactions (without added dextran sulphate). The table shows the numbers of positive PMCA reactions after up to 6 rounds of serial PMCA. All buffy coat samples gave positive results, while only 4/14 reactions seeded with plasma were positive. (DOCX) [file ppat.1009276.s007.docx]

**S7 Table**

| **Sheep group** | **Number of sheep tested** | **Number positive by PMCA/number tested** | |
| --- | --- | --- | --- |
|  |  | **Buffy coat** | **Plasma** |
| Donors (oral infection) | 4 | 4/4 | 0/4 |
| Recipients (intravenous infection) | 10 | 10/10 | 4/10 |
| BSE-exposed donors, infection not confirmed (negative controls) | 2 | 0/2 | 0/2 |
| Negative control donors (mock-infected) | 2 | 0/2 | 0/2 |
