# Supplementary figures and images for: Preclinical transmission of prions by blood transfusion is influenced by donor genotype and route of infection
Source: PLoS Pathog. 2021 Feb 18;17(2):e1009276. doi: 10.1371/journal.ppat.1009276 (PMC7891701; doi:10.1371/journal.ppat.1009276)

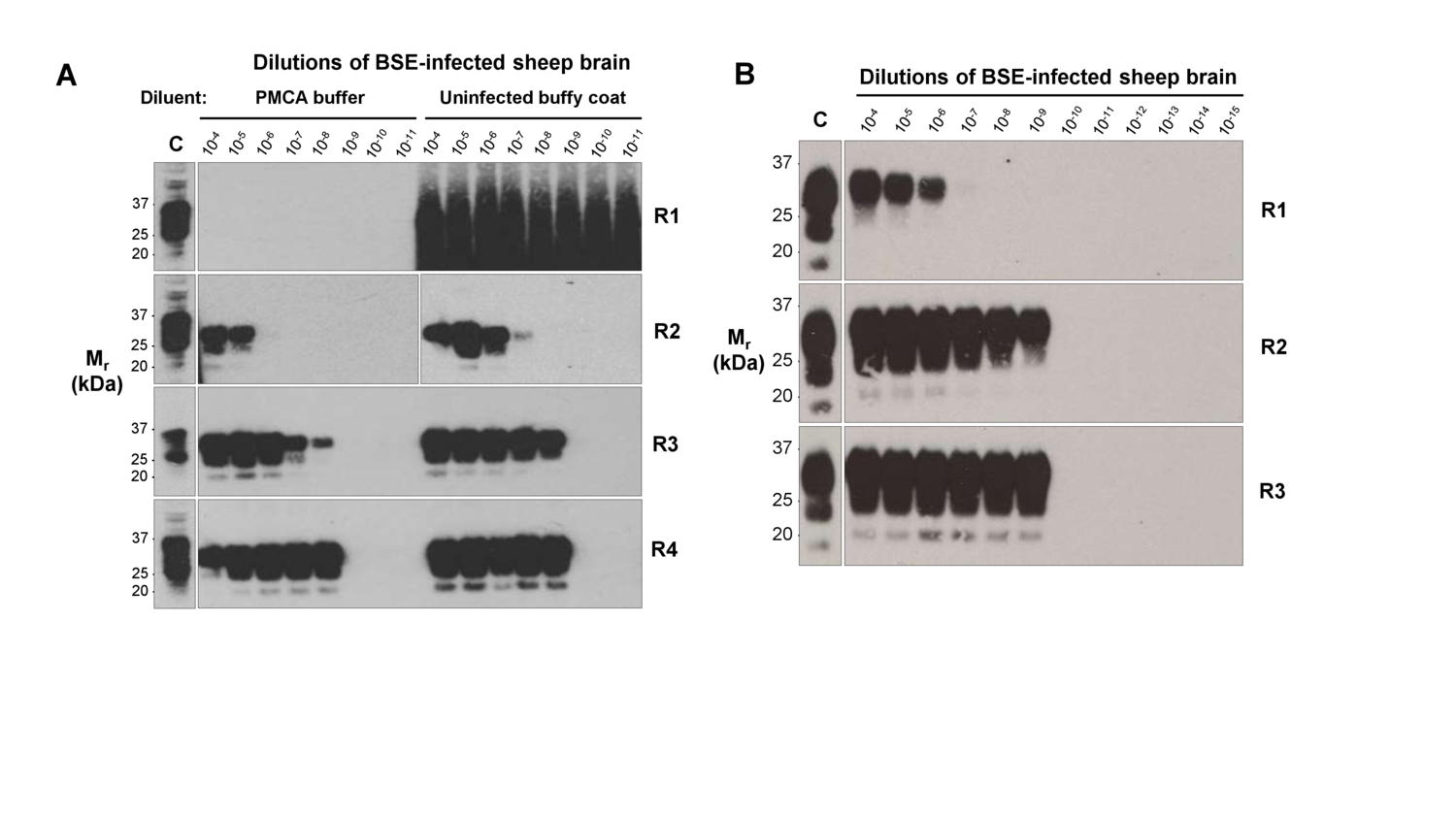

Supplement: S1 Fig — A. To assess the potential inhibitory effects of blood components, PMCA reactions were seeded with tenfold serial dilutions (10−4 to 10−11) of BSE-infected sheep brain homogenate diluted in either PMCA buffer or uninfected buffy coat. The limit of detection (brain dilution of 10−8) was the same for both dilution series, and was reached after 3 rounds (R3) of serial PMCA. C–positive control; BSE-infected sheep brain homogenate (no PK digestion; 0.36mg brain equivalent). B. Dextran sulphate was added to give a final concentration of 0.5% (w/v) in PMCA reactions seeded with tenfold serial dilutions of the same BSE-infected sheep brain homogenate. The limit of detection (brain dilution of 10−9) was reached after 2 rounds (R2) of PMCA in the presence of dextran. C–positive control; BSE-infected sheep brain homogenate (PK digested; 1.7mg brain equivalent). (TIF) [file ppat.1009276.s008.tif]

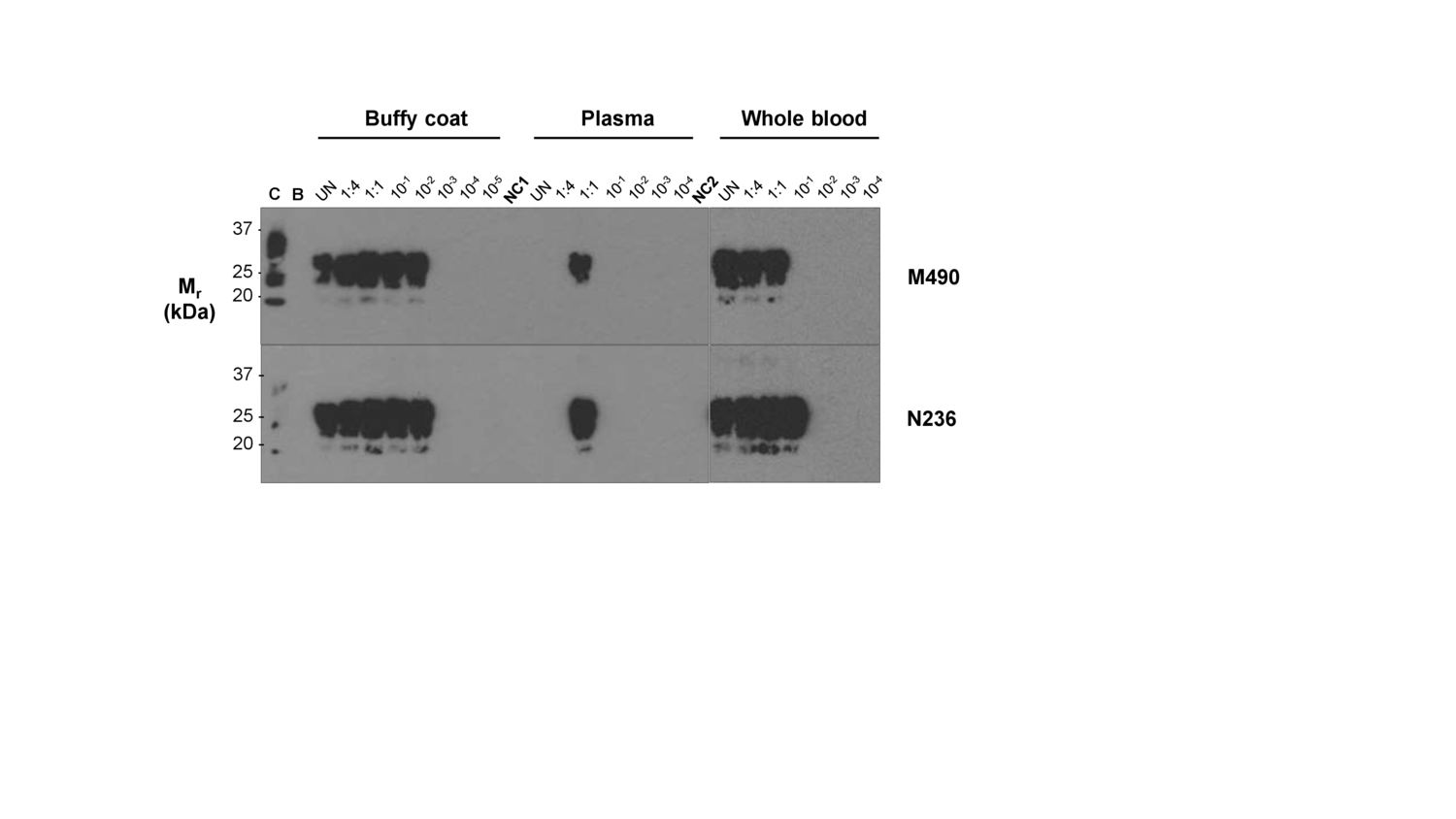

Supplement: S2 Fig — Samples of whole blood, buffy coat and plasma from two BSE-infected donor sheep (M490 –upper panel; N236 –lower panel) at the clinical stage were used to seed PMCA reactions either undiluted (UN) or following dilution in PMCA buffer as indicated (1:1, 1:4, tenfold serial dilutions). The image shows the results after 6 rounds (R6) of serial PMCA (no added dextran), demonstrating positive amplification in all blood fractions with varying sensitivity. C–positive control; scrapie-infected Rov9 cell lysate (PK digested; 37.5 μg total protein equivalent). B–blank lane. NC1, NC2 –negative control samples (buffy coat and mesenteric lymph node, respectively) from mock-infected sheep. (TIF) [file ppat.1009276.s009.tif]
