# Supplementary material for: Preclinical transmission of prions by blood transfusion is influenced by donor genotype and route of infection
Source: PLoS Pathog. 2021 Feb 18;17(2):e1009276. doi: 10.1371/journal.ppat.1009276 (PMC7891701; doi:10.1371/journal.ppat.1009276)
Supplement: Attachment — Submitted filename: Response to reviewers.docx [file ppat.1009276.s010.docx]

24^th^ December 2020

PLoS Pathogens,

Editorial Office,

San Francisco,

California,

USA.

**PPATHOGENS-D-20-02404 - Response to Reviewers**

**Title: Preclinical transmission of prions by blood transfusion is influenced by donor genotype and route of infection**

Dear Editors,

Thank you for your consideration of our manuscript. We would like to thank the reviewers for their positive evaluation of the paper, and the promptness and thoroughness of their reviews.

The following letter details our revisions and responses to the reviewer’s comments and recommendations. The line numbers refer to the copy of the manuscript marked via Track Changes, for ease in identifying the modifications to the text.

**Reviewer #1**

1) “No IHC data is shown, negating the need for a methods section.”

- I have now included IHC and Western Blot results in Supplementary File 2, so have retained the methods.

2) “Clearly denote the number of rounds required for PMCA detection throughout the manuscript, including abbreviations, i.e. (R) used in some cases, Round in others.”

- The number of PMCA rounds is clearly indicated in all figure legends – I have made slight amendments (e.g. lines 839 & 857 of marked manuscript) for consistency.

3) “Lines 279 and 280: Each line mentions ‘this figure’, yet I believe the authors are referring to Table 1. Please clarify.”

- “This figure” actually referred to the transmission rate (mentioned in the previous sentence). The wording has been amended for clarity in response to this comment, and issues raised by Reviewer #3 – see line 309.

4) “Lines 298-303: Refers to a BSE brain mouse bioassay titration. Please provide data or publication reference, as this titration was used to estimate infectious doses for blood components.”

- I have included a table (Table S3), in Supplementary File 1, with the results of the pilot titration of sheep BSE in TgShpXI mice. These results were not actually used to calculate the infectious titres in blood components, as incubation periods were outside the range where there is a linear relationship with infectious dose – instead we used the limiting dilution method used by Paul Brown and Bob Rohwer to calculate titres. I have added a sentence to clarify this (lines 337-339).

5) “Tables 1 and 3. For consistency please include % Neg.”

- Tables have been amended.

6) “Line 354: “At the time of blood collection, it was not possible to determine which of the primary recipients acting as donors in this experiment were infected with BSE”. Verifying that a RAMALT biopsy was not collected to provide scrapie PrPSc deposition status.”

- The blood collections from primary recipients were carried out between November 2008 and July 2009 – most of the publications relating to the use of RAMALT came out at this time. Therefore RAMALT biopsy had not been part of the original plans for the project – it was later incorporated when we secured additional funds and amendments to our Project Licence, but the first biopsies were not performed until 2011.

7) ‘Line 391: “The most likely explanation for these results is that the probability of transmission is related to the stage of the incubation period reached by donor sheep at the time of blood collection”
   a. Please clarify what is meant by “stage of incubation period”. Do the authors mean infectivity titers in blood may be too low to initiate infection, or that prions may be absent from blood all together?
   b. PMCA blood analysis did not include dilutional series, thus cannot provide information about titers in blood. Thus could lines 391 and 398 suggest again titer vs stage of disease?’

- In response to a), and comments of Reviewer #3, I have clarified this by including a definition of “stage of incubation/survival period” in the text – lines 437-438 (it is also in the legend for Figure 2). I have also reworded the final sentences in this paragraph to try to make the meaning clearer, and include an alternative explanation (lines 444-448). Throughout this section, when I refer to blood titres, I am to some extent speculating on possible explanations for the observation that codon 141 genotype appears to influence the probability of transmission. Since the mouse bioassay turned out not to be sensitive enough to estimate the actual infectivity titres, this is not based on data. I agree that PMCA results do not provide information about titres, as PMCA detects PrP^Sc^ rather than infectivity.

8) “Fig. Legends and Fig. Titles: Seeding activity vs PrPsc. Please address for consistency.”

- Figure legends and titles have been amended to PrP^Sc^ rather than seeding activity (lines 832, 843, 853).

**Reviewer #2**

“The blood samples for transfusion were taken at 10 months post infection from donors and 15 months post infection from primary recipients. PMCA results show that blood samples of only 6/10 donors (orally infected with BSE) were positive, whereas after 12 months 9/10 and after 18 months 10/10 were positive. In secondary transfusion, blood samples for transfusion was obtained at 15 months, a time point when all samples that were analysed were positive in PMCA. Therefore, the authors should consider adding to the discussion whether the sheep infected by transfusion are indeed more likely to transmit infection, as presumably at 15 months post infection, transfusion of blood from orally infected sheep might have resulted in 100% transmission.”

- I agree that the PMCA data would suggest that donors would have had similarly high transmission rates to primary recipients if blood had been collected at 15 months post-infection. However, I tried to make the point in the discussion, that higher transmission rates for primary recipients could also be predicted at much earlier preclinical time points, also based on the PMCA data. Therefore, the period over which sheep infected by transfusion are potentially infectious is, on average, much higher than for sheep infected orally. I have amended the Discussion to make this point more explicit (lines 610-613).

**Reviewer #3**

“The authors mentioned lines 265-267 that “The majority of donor sheep (33/39) orally dosed with BSE-infected brain homogenate developed typical clinical signs, and were confirmed as being infected by detection of PrPsc in tissues tested post mortem by Western blotting and/or immunohistochemistry (IHC)”. They announced that brain, tonsil, spleen, Peyer’s patches, mesenteric lymph nodes and prescapular nodes were culled. Did the authors test all the samples for all the animals, including for the negative ones? The authors also defined “evidence of BSE infection” as “clinical signs and/or positive results from western blotting and IHC” (lines 277-278). The criteria of inclusion are clearly unclear, since they may literally include several situations, ranging from a sheep under incubation with only one positive follicle to clinically-affected animals at the terminal stage of the disease.”

- I have amended the table in Supplementary File 2 - this is now 3 separate tables (Tables S5 – S7), which show the results of IHC and Western blotting on the tissues of individual animals, as well as the clinical status of the animals at the time they were culled/died, for donors, primary recipients and secondary recipients respectively. From this it may be seen that, even when sheep did not show clinical signs of BSE, those identified as BSE-infected had detectable deposits of PrP^Sc^ in both brain and (almost always) at least one lymphoid tissue, indicating that they were in the late preclinical phase of infection. I have also amended the text to give more explanation of clinical outcomes and the results of IHC/Western blotting (lines 265-308; 395-396; 403-406).

“Moreover, incubation period is defined as the interval between the date of infection and the date on which the animal was culled, and thus include the clinical period that is very different upon animals and dependent on the moment the authors decided to proceed to the euthanasia. What is the duration of the clinical period for each animal? Based on the pattern of information provided in this manuscript, it is impossible to draw comparisons between animals according to their status (Infection? Disease?) and their incubation period, which is a capital information in prion diseases since it is reputed to be proportional to the initial infectious dose. For each animal presented in this study, a detailed table should be provided with individual information about clinical signs and biochemical / IHC positivity in all the tested organs.”

- I agree that according to a strict definition of “incubation period”, it should reflect the interval between the date of infection and the date of clinical onset, rather than date of culling. The reason that we did not use this definition is that the onset of disease can be very subtle and variable between sheep, and it is thus highly dependent on the experience and observation skills of the person performing the clinical scoring. This was a very long term project, and several different members of staff were involved in clinical scoring over time, and so the reliability and consistency of detection of disease onset is likely to be quite variable. By contrast, we had a well-defined scoring system for defining clinical end points (as required by the terms of our Home Office Project Licence), so that animals were culled as soon as it was clear that they had clinical signs consistent with BSE, but before they reached advanced stages of disease that could severely compromise their welfare. Therefore we consider that the interval between infection and culling gives the most accurate reflection of variation in incubation period, and this has been the accepted definition in all our previous publications for this project, and others involving experimental infection of sheep with TSEs. However, in response to this point, I have changed references to incubation period throughout the manuscript to “survival period” instead, which more accurately reflects the data presented.

“Moreover, information should be provided upon the cause of death for negative animals, including the three animals mentioned as ataxic (line 268).”

- The additional information presented in Table S5 – S7, now includes the recorded clinical status or cause of death for all the sheep in the project. There were a number of deaths from various causes, e.g. injury, neoplasia, urolithiasis, dental issues, weight loss etc, which are all recorded as “intercurrent”. We also had a number of sheep that presented with signs of ataxia, and were thus initially scored as clinically positive for BSE, but showed no evidence of PrP^Sc^ deposition in tissues by IHC/Western blot. In three of these sheep examined by a neuropathologist (Martin Jeffrey) there was evidence of axonal degeneration affecting the spinal cord, which he considered most likely to have a toxic or metabolic cause. However, further biochemical and haematological testing failed to establish a definitive cause, and the cases were therefore classified as “idiopathic ataxia”. These cases mostly occurred over a distinct time period in the project (2008-2009) and were not seen later when the sheep were moved from Compton to Edinburgh, further suggesting that there could be an environmental or nutritional cause. In Tables S5 – S7, their clinical status is recorded as “Ataxia”, and the text in the Results section has been amended to explain this (lines 276-285). Some recipients were also identified as having atypical scrapie, which can be easily distinguished from BSE – this is also explained in the text (lines 302-308) and these cases are indicated (AS) in Tables S6 & S7.

“The authors mentioned the occurrence of altered clinical phenotypes in a macaque model of transfusion (line 501); did they observe similar observations in this sheep model?”

- Although it is possible that the cases of “idiopathic ataxia” mentioned above are similar to the altered clinical phenotype seen in the macaques, we think this is unlikely because cases occurred both in orally infected sheep and sheep infected by transfusion, whereas in macaques the altered clinical phenotype was only found in transfusion recipients, as far as I am aware. Clinical cases of ataxia in the absence of PrP^Sc^ deposits in brain and lymphoid tissue detectable by IHC/Western blot have not previously been reported in several studies where sheep were orally infected with BSE. I have made a comment on this in the Discussion (Lines 560-562 ).

“As mentioned above, incubation period is expected to be proportional to the initial infectious dose. In this study the relation between these two parameters is apparently incoherent since bioassay in transgenic mice allowed the estimation of infectivity titers, which is completely unrelated to the incubation period in sheep: for example, buffy coat of N236 is estimated 13.1 ID/ml and transmit disease in 904 days, whereas buffy coat of N257 is estimated 2.8 ID/ml and transmit disease in 639 days.”

- Although it is generally true that incubation period is proportional to infectious dose, I have only ever seen this reliably applied in rodent studies, in which a parallel titration of the prion strain in question is carried out in the relevant experimental model to determine the relationship between dose and incubation period. In the context of this study, this would have involved transfusing groups of sheep with serial dilutions of BSE-infected blood, which was not feasible due to the expense of such a large additional experiment (although we did consider it). Also, the incubation periods in an outbred population such as sheep are much more variable than in inbred laboratory rodents – this can be seen by the large standard deviations when calculating mean survival periods of the donor sheep, which all received the same infectious dose (lines 272-275). An additional complication in this experiment was the unanticipated effect of the *PRNP* codon 141 polymorphism on incubation period. For these reasons, I have deliberately not made any inferences about infectious dose from the incubation/survival periods in sheep, nor do I think it is justified to do so. This was the reason for using the transgenic TgShpXI mouse bioassay for estimation of blood titres, but unfortunately it was not sensitive enough to provide data on the majority of blood samples.

“The authors mentioned that all the samples were tested by PMCA; a reliable comparison between bioassay in mice, PMCA and transmission to sheep would have been expected. Moreover, BSE was transmitted to several sheep after plasma transfusion: the authors should discuss why no bioassay in mice was positive with plasma samples.”

- Not all samples were tested by PMCA, this is clear in the text and table/figure labelling, which states that buffy coat samples from selected time points were used (because the sensitivity of detection was greatest in buffy coat) – see lines 462-468, 488.
- Table 5 shows the comparison between PMCA results on buffy coat samples and transmission to sheep – I have added a footnote to indicate that the 5 buffy coat samples that transmitted infection to mice were included in the PMCA analysis. This is as far as we can go in comparing the results of bioassay, PMCA and transmission to sheep, because the sensitivity of the mouse bioassay in detecting BSE infection in sheep blood samples was disappointingly low. This is also the reason why there were no positive bioassay results with plasma (or RCC) samples – presumably the titres in plasma and RCC were below the levels of detection for the bioassay. I have amended the text slightly to make this clearer (lines 348-355).

“One main conclusion of the paper relies on the efficiency of leucodepletion: the authors underline the significant differences between the rates of BSE transmission after transfusion of blood products (plasma, platelet and RBCC) and their leukodepleted counterparts. However, one could have another lecture of these results. Indeed, when transmission did not occur with non-leucodepleted blood products (plasma for N245, N189, N204, N231, N157, N180; platelets for N231 and N157; RCC for N189, N204 and N231), the absence of transmission after leucodepletion is also expected. Thus, one may focus on the effect of leucodepletion only for blood products that transmitted infection, and the efficiency of leucodepletion would be analyzed for paired subjects.
In this context, the analysis of leucodepletion on plasma is limited to donors N233, N261 and N232. Leucodepletion was efficient only on N232 plasma (no transmission after leucodepletion), whereas leucodepletion effect was weak (N261, doubling of incubation period) to null (N233, same incubation periods) for the two other plasmas. For RCC (limited to N233, N261, N232, N157 and N181), leucodepletion was efficient for only two samples (N157 and N180 donors), but inefficient for the three other ones (N233, N261 and N232). For PLT, whereas N261 should not be considered (recipient of leukodepleted PLT died after 33 days), leucodepletion seemed efficient since five recipients among 6 did not develop disease.
According to their results, the authors should modulate their conclusions on the efficiency of leucodepletion, that is finally mostly focused on platelets, and they should discuss these differences of efficiency; do the authors think that these differences might be linked to the plasmatic soluble infectivity that would be differently distributed among the types of blood products?”

- I agree that the most meaningful comparison of the effect of leucodepletion is for recipients of matched non-leucodepleted and leucodepleted components from the same donor, where the non-leucodepleted component transmitted infection – this is shown in Table 3. As the reviewer points out, the numbers in each group are very small – this meant that in the statistical analysis, a significant effect of leucodepletion was only evident by combining the data for all three components. For individual components, even platelets, there was insufficient power for statistical tests to demonstrate significant differences between the non-leucodepleted and leucodepleted groups. Therefore, although I had also noted that leucodepletion of platelets appeared most efficient in preventing transmission, I did not feel that more definitive conclusions could be supported by the data. I believe that a plausible explanation for the platelet results, is that the non-leucodepleted platelets are likely to have higher levels of WBC contamination because of their method of preparation. I have added a comment to this effect in the Discussion (lines 533-539).

I trust that these revisions are satisfactory – if you have any further questions or matters requiring clarification, please let me know.

Yours faithfully,

Fiona Houston, BSc, BVMS, PhD (corresponding author).

Group Leader,

Division of Infection and Immunity,

The Roslin Institute.

Email: Fiona.houston@roslin.ed.ac.uk
